# Transcription factor E2F4 is an indicator of poor prognosis and is related to immune infiltration in hepatocellular carcinoma

**DOI:** 10.7150/jca.51616

**Published:** 2021-01-21

**Authors:** Qiuxian Zheng, Qiang Fu, Jia Xu, Xinyu Gu, Haibo Zhou, Chen Zhi

**Affiliations:** 1 State Key Laboratory for Diagnosis and Treatment of Infectious Diseases, National Clinical Research Center for Infectious Diseases, Collaborative Innovation Center for Diagnosis and Treatment of Infectious Diseases, The First Affiliated Hospital, College of Medicine, Zhejiang University, Hangzhou 310003, China.; 2 School of Continuing Education, Zhejiang University, Hangzhou 310003, China.

**Keywords:** HCC, E2F4, hub genes, prognosis, immune infiltration

## Abstract

**Background:** Recent studies have shown that the transcription factor E2F4 is involved in the progression of various tumors, but its expression and influence on immune cell infiltration and biological functions are largely unknown in hepatocellular carcinoma (HCC).

**Methods:** The Cancer Genome Atlas (TCGA) database, the Tumor Immune Estimation Resource (TIMER) and related online tools as well as a tissue microarray (TMA) were used for analyses in our study.

**Results:** E2F4 expression was elevated in HCC tumor tissue compared with adjacent normal tissue at both the mRNA and protein levels. Overexpression of E2F4 was markedly related to a poor prognosis in HCC patients. In addition, positively and negatively correlated significant genes of E2F4 were identified in HCC. Pathway enrichment analyses revealed that the top 100 positively correlated significant genes of E2F4 were closely related to nuclear splicing and degradation-related pathways. Furthermore, nine hub genes correlated with E2F4 expression were validated based on a protein-protein interaction (PPI) network. It was also demonstrated that E2F4 expression was negatively correlated to immune purity and positively correlated to immune cell infiltration.

**Conclusion:** E2F4 could serve as a novel biomarker for HCC diagnosis and prognosis prediction.

## Introduction

Hepatocellular carcinoma (HCC) has become the third-leading cause of cancer-related mortality worldwide^1^, ^2^, with the devastating complication of chronic liver disease, due to the difficult detection of HCC during the early stages when the disease is curable 3. Many patients with advanced HCC have rapid cell proliferation, metastasis, and insensitivity to chemotherapy with an abysmal prognosis. Consequently, the majority of HCC patients are diagnosed after the tumor has grown to a considerable size 4. Therefore, novel biomarkers provide an approach for early detection, which is urgently needed to improve the prognosis of HCC patients.

E2F, a group of genes encoding a transcription factor protein family, act as regulators of cell differentiation, response to DNA damage, and cell life cycle 5, 6. It has been shown that compared to normal tissues, E2F family genes are highly expressed in several types of tumor tissue, including HCC 7. The E2F family is one of the critical tumor-suppressor/oncogene regulators are also involved in regulating telomerase expression and drug sensitivity 8, 9. Hypo-methylation, amplification, and tumor protein p53 (TP53) mutations have a close relationship with the E2F family genes in many cancer types including HCC^6^, ^10^. As a well-characterized potent transcriptional inhibitor and E2F family member, E2F4 is a crucial factor that promotes tumor growth via recruiting pocket proteins, binding with retinoblastoma (RB) family proteins and functioning with histone-modifying enzymes^11^, which is crucial for the regulation of differentiation and cell cycle arrest^12^. Certain evidence has illustrated that E2F4 is highly enriched in leucine/isoleucine-rich hydrophobic nuclear export signals, which cannot activate E2F-responsive genes *in vivo* 13. Researchers have shown that E2F4 is involved in the tumorigenesis of Burkitt lymphoma 14, cervical cancer 15, colorectal cancer 16, and acute myeloid leukemia 17. However, the expression and specific roles of E2F4, including the protein regulation network, remain unclear in HCC.

A growing body of evidence also shows that E2F4 is remarkably correlated with tumor immune cell infiltration 18. E2F4 functions as an inducible protein in the immune response of eukaryotes. In mammals, E2F4 is a critical member of transcriptional factors involved in innate immune responses, such as expression of toll-like receptor 8 (TLR8) and CD14, and the downstream signal transducer and activator of transcription (STAT1) pathway 18. However, as an immuno-suppressive protein, the regulatory function of E2F4 in HCC remains unknown.

To enhance our understanding of E2F4 crosstalk with immune cells, we determined the relationship between E2F4 and programmed death-ligand 1 (PD-L1) expression, and also validated the relationship between E2F4 gene expression and immune cell infiltration. We analyzed the HCC cohort from the TCGA dataset using multiple online resources. We also investigated the expression level, clinical characteristics, mutations, and differentially expressed genes (DEGs) of E2F4. With these data, we assessed the signaling pathways of DEGs and contrasted the protein-protein interaction (PPI) network to identify hub genes. Finally, the expression of E2F4 in HCC and its association with immune cell infiltration were also explored.

## Materials and Methods

### Data collection and processing

The RNA sequencing (RNA-seq) and corresponding clinical data from pan-cancer patients as well as HCC patients were downloaded from The Cancer Genome Atlas Liver Hepatocellular Carcinoma (TCGA-LIHC) data collection (https://xenabrowser.net/datapages/) as previously described 19. Three hundred seventy-four liver cancer tissues and 50 adjacent tissues were included in the HCC patient cohort. E2F4 gene expression profiles were further analyzed by characteristics according to age, race, tumor grade, tumor stages, TP53 gene mutation status, and lymph node metastasis status. E2F4 gene expression was determined in pan-cancers and HCC patients. The pan-cancer cohort gene profiles were also downloaded from the publicly available TCGA database, including tumor and matched non-tumor tissues. The E2F4 gene expression level among various diseases was then estimated. The expression of E2F4 across the pan-cancer data was assessed via the UALCAN website (http://ualcan.path.uab.edu/) 20.

### Overall clinical survival of E2F4 gene-related HCC patients

The survival curves of HCC cohort patients were analyzed online via The Kaplan Meier plotter (https://kmplot.com/) based on TCGA analysis as described previously 21, 22. The overall survival (OS), recurrence-free survival (RFS), progression-free survival (PFS), and disease-specific survival (DSS) were the designated endpoints. Patients were divided into low and high expression groups based on the median expression of E2F4. Comparisons were made between the two groups for each type of survival curve.

### Mutation types of E2F4 gene

The gene mutations and co-expression of E2F4 were computed and analyzed using CBio-Cancer Genomics Portal (cBioPortal) databases (https://www.cbioportal.org/) 23. Searching the term “E2F4” enabled the acquisition of the full mutation distribution across all tumor and non-tumor tissues. After selecting “HCC”, the mutation frequencies and mutation types that occur in HCC samples were obtained.

### E2F related immune infiltration

Tumor Immune Estimation Resource (TIMER, https://cistrome.shinyapps.io/timer) is a database resource for systematic evaluations that contains information regarding the clinical impact of immune infiltration among various types of cancer 24. Immune cells, such as B cells, T cells, neutrophils, and antigen-presenting cells (APCs) were included. The microenvironment in tumor tissues was estimated via a novel statistical algorithm. Furthermore, considering that TP53 is the most frequently mutated gene in HCC, the correlation between TP53 mutation status and immune cell infiltration was analyzed via the TIMER website.

### Identification of differentially expressed genes

LinkedOmics (http://www.linkedomics.org/) contains various clinical data for pan-cancer types, and all patients were included as described in a previous study 25. To determine candidate biomarkers, gene expression profiles were analyzed. To further identify DEGs, associations between billions of attribute pairs for each cancer cohort were analyzed and visualized. The top 10 positively and negatively co-related genes were selected.

### E2F4-related hub gene selection and validation

The PPI network and module analysis were performed for further exploration. The top nine genes identified by degree were considered hub genes and were noted for further study. Additionally, the Gene Expression Profiling Interactive Analysis 26 (GEPIA, http://gepia.cancer-pku.cn/) and Cytoscope software (https://cytoscape.org/) were used to determine hub genes and investigate the correlation between hub genes and E2F4 expression.

### Pathway network construction

To decipher the potential molecular mechanisms and explore the functional annotation of E2F4-related DEGs in HCC genomic profiles, Gene Ontology (GO) and Kyoto Encyclopedia of Genes and Genomes (KEGG) enrichment analyses were performed.

### Clinical tissues

Tissue microarray (TMA) containing 90 HCC and 90 normal liver tissues was purchased from Outdo Biotech (Shanghai, China). All of the clinical information including age, gender, grade, TNM stage, as well as survival time, was collected and the details are presented in **Table 1**.

### Immunohistochemistry (IHC) analysis

The IHC staining pattern of E2F4 was evaluated. Briefly, after incubation with the E2F4 primary antibody (dilution 1:300, [EPR8259] (ab150360) Abcam, Cambridge, MA), the tissue microarray was probed with biotinylated goat anti-rabbit secondary antibody rabbit IgG, (Dilution: 1:10,000, Cell Signaling Technologies, Inc.), and the remainder of the process was carried out according to a previously published study 27.

### Statistical analysis

A *t*-test with a p value < 0.05 was applied to identify significance with statistical differences. The OS analysis was performed using R programming of “survival” and “survive” packages online. The OS, RFS, PFS, and DSS were separated and calculated by the median gene expression level of E2F4. Spearman's correlation was applied to identify the relationship between E2F4 and associated genes and pathways. A p value < 0.05 was used to identify significant differences.

## Results

### E2F4 was highly expressed in pan-cancers, and the E2F4 gene was differently expressed according to the clinical characteristics of HCC

**Figure 1:**
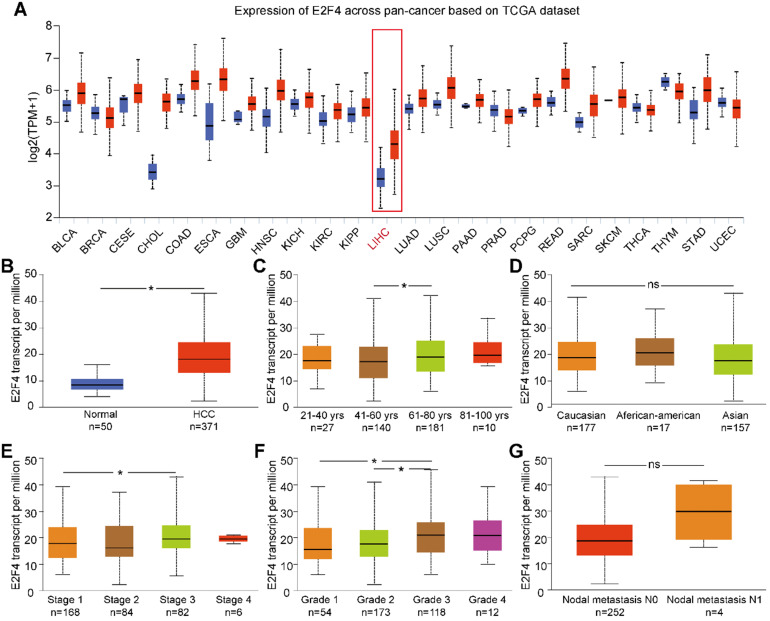
** E2F4 gene expression and the clinical characteristics associated with E2F4 expression and clinical stage based on data from TCGA database. (a)** Comparison of E2F4 expression between different cancer types and normal tissues in the TCGA dataset. **(b)** E2F4 expression in HCC tumor tissues compared to non-tumor tissues. **(c)** E2F4 expression in HCC tumor tissues among different age groups. **(d)** E2F4 expression in HCC based on different races. **(e)** E2F4 expression based on the tumor stage in HCC. **(f)** Comparison between different HCC tumor grades and E2F4 gene expression. **(g)** E2F4 expression with nodal metastasis N0 and N1. Chi-square test analysis was applied in this study and we recognised p value <0.05 as statistically significant. **Abbreviations:** LIHC, liver hepatocellular carcinoma; HCC: hepatocellular carcinoma; TCGA: The Cancer Genome Atlas; TPM: transcripts per million.

UALCAN was used to analyze the expression of E2F4 mRNA from the pan-cancer data. The results showed that the transcription of E2F4 was increased in most cancers (**Figure 1A**). Additionally, the transcriptional level of E2F4 in the TCGA-LIHC cohort was significantly higher compared with normal tissue (**Figure 1B**). To further investigate the levels of E2F4 mRNA in HCC patients, the clinicopathological characteristics of HCC patients were analyzed. In this study, a slight but statistically significant difference was observed between stages 1 and 3. In regards to the tumor grade, significant differences in E2F4 transcription were found between both grade 1 and grade 2 when compared to grade 3 (all p < 0.05; **Figure 1E and 1F**). However, the E2F4 mRNA showed no significant differences in regard to age, race, or node metastasis status among patients with HCC (**Figure 1C, 1D and 1G**). These findings suggest that E2F4 expression was upregulated in HCC tissues, and it has potential to be a novel biomarker for HCC diagnosis.

### Elevated E2F4 expression impacts the clinical prognosis of patients with HCC

**Figure 2:**
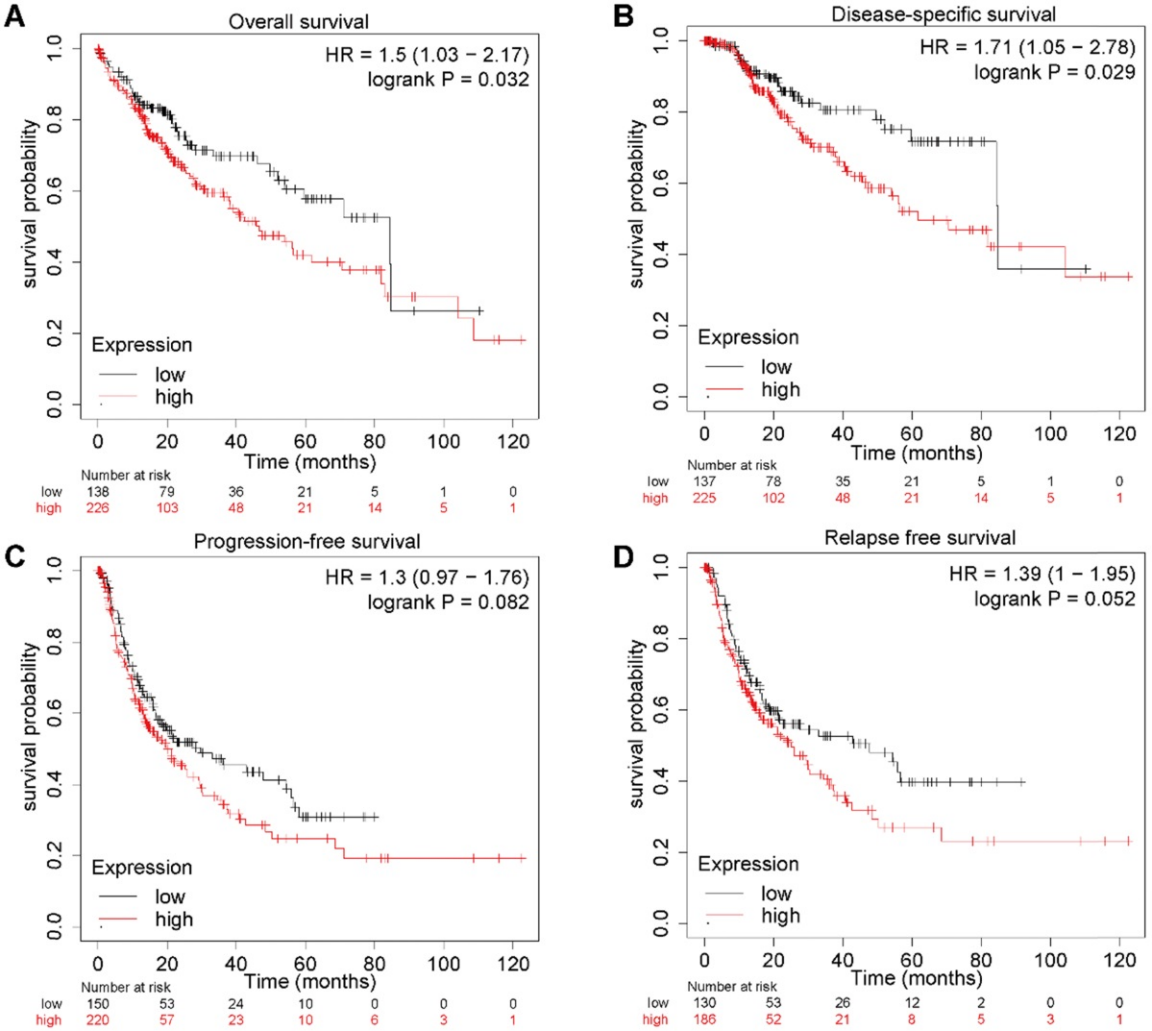
**E2F4 gene expression and clinical outcomes. (a)** OS curves showing stratification of HCC patients in the TCGA dataset based on E2F4 expression levels. **(b)** DSS curves of patients with either high or low E2F4 gene expression. **(c)** PFS curves of patients with either high or low E2F4 gene expression. **(d)** RFS curves of patients with either high or low E2F4 gene expression. **Abbreviations:** OS, overall survival; TCGA, The Cancer Genome Atlas; DSS, disease-specific survival; PFS, progression-free survival; RFS, relapse-free survival.

To further explore the relationship between E2F4 expression and clinical outcomes in patients with HCC, a Kaplan-Meier plot was used to evaluate patient prognosis in relation to different E2F4 expression levels. The OS of HCC patients with high E2F4 expression was significantly worse than those with low E2F4 expression (hazard ratio [HR] = 1.5 [95% confidence interval (CI): 1.03-2.17], log rank p = 0.032; **Figure 2A**). Moreover, patients with high expression of E2F4 had worse DSS (HR = 1.71 [95% CI: 1.05-2.78], log rank p = 0.029; **Figure 2B**). There were no significant differences in PFS (HR = 1.3 [95% CI: 0.97-1.76], log rank p = 0.082; **Figure 2C**) or RFS (HR = 1.39 [95% CI: 1-1.95], log rank p = 0.052; **Figure 2D)**. Collectively, elevated E2F4 expression was significantly related to a worse prognosis for patients, indicating the E2F4 may be able to serve as a prognostic factor.

### E2F4 expression was increased in HCC tissues and related to poorer prognosis of patients

**Figure 3:**
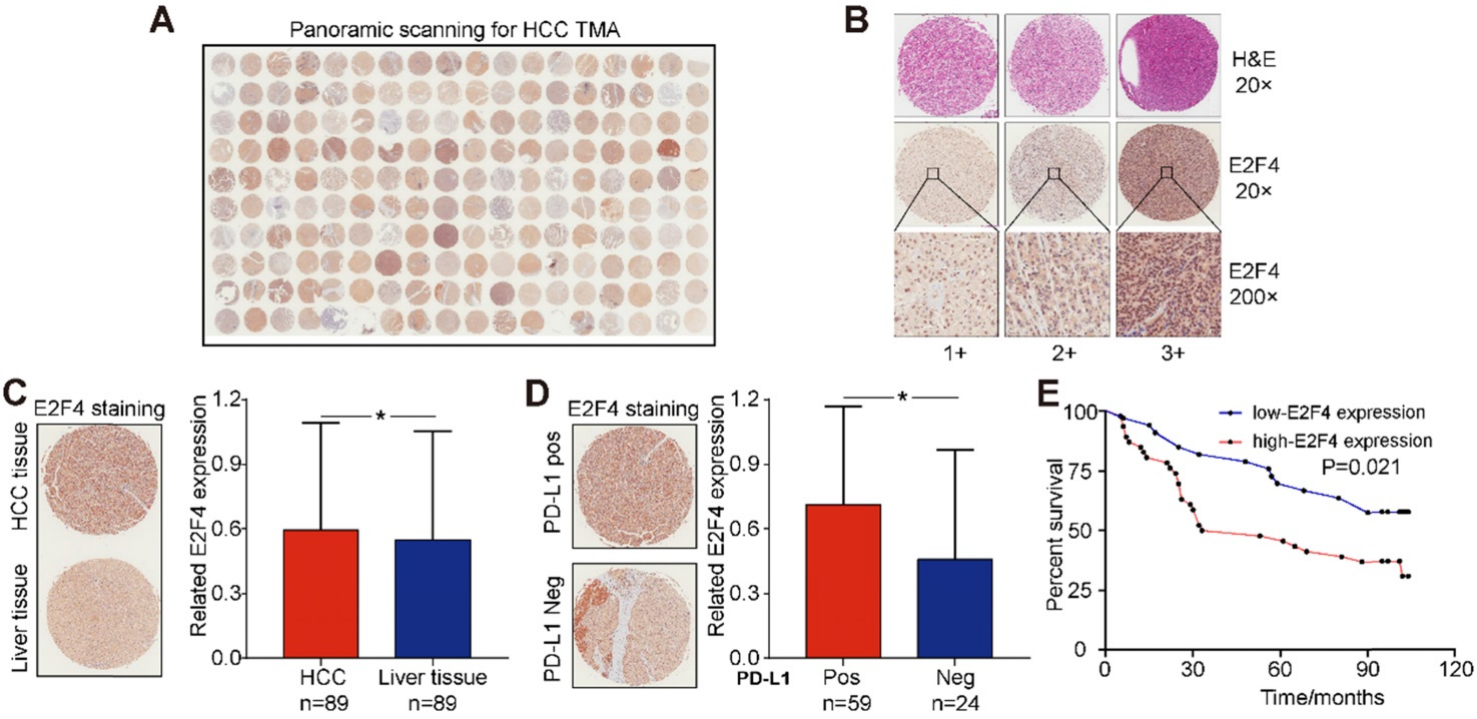
**E2F4 expression was elevated in HCC tissues and high E2F4 expression was associated with poorer OS. (a)** Representative E2F4 staining in HCC and normal tissues. **(b)** Representative E2F4 and immunohistochemical staining patterns with different staining scores in HCC tissues. **(c)** Chi-square test analysis was applied, and p value <0.05 was identified as statistically significant. Expression of E2F4 protein in HCC tissue compared to normal liver tissue. (p = 0.036). **(d)** Chi-square test analysis. P value <0.05 is statistically significant. Association between up-regulated E2F4 expression and positive PD-L1 staining. (p = 0.029, χ^2^=4.751). **(e)** E2F4 expression associated with prognosis. **Abbreviations:** OS, overall survival; HCC, hepatocellular carcinoma; PD-L1, programmed death-ligand 1.

The association between E2F4 protein expression and clinical characteristics of patients with HCC were assessed. Representative micrographs reveal E2F4 IHC staining patterns with different staining scores in HCC and paired normal liver tissues, and the staining patterns were categorized into three groups, 1+, 2+ and 3+, according to staining intensity (**Figure 3A and 3B**). As shown in the Figure 3C, E2F4 expression was increased in HCC tissues when compared to the paired liver tissues (p = 0.036, **Figure 3C**), which was consistent with the results from the TCGA cohort (**Figure 1B**). In addition, we found that elevated E2F4 expression was positively correlated with the PD-L1 positive subgroup (p = 0.029, **Figure 3D**). There was no significant correlation among E2F4 expression and age, gender, grade, TNM stage, cirrhosis, or alpha-fetoprotein (AFP) levels (**Table 1**). Moreover, we assessed the correlation of E2F4 expression with survival of patients with HCC. Kaplan-Meier curves revealed that patients with increased E2F4 expression had shorter OS times compared with those who had reduced E2F4 expression (**Figure 3E**). Univariable and multivariable Cox regression analyses were adopted to determine the risk factors for OS prognosis. Univariable analysis demonstrated that advanced grade, late TNM stage, and high E2F4 expression were potential risk factors for decreased OS in HCC. Through further analysis, high E2F4 expression was found to be an independent predictor of OS according to multivariable analysis (**Table 2**). Taken together, these findings demonstrated that up-regulated E2F4 expression could service as a novel predictor for prognosis in HCC.

### The mutation characteristics of E2F4 and the correlation of mutated genes

**Figure 4:**
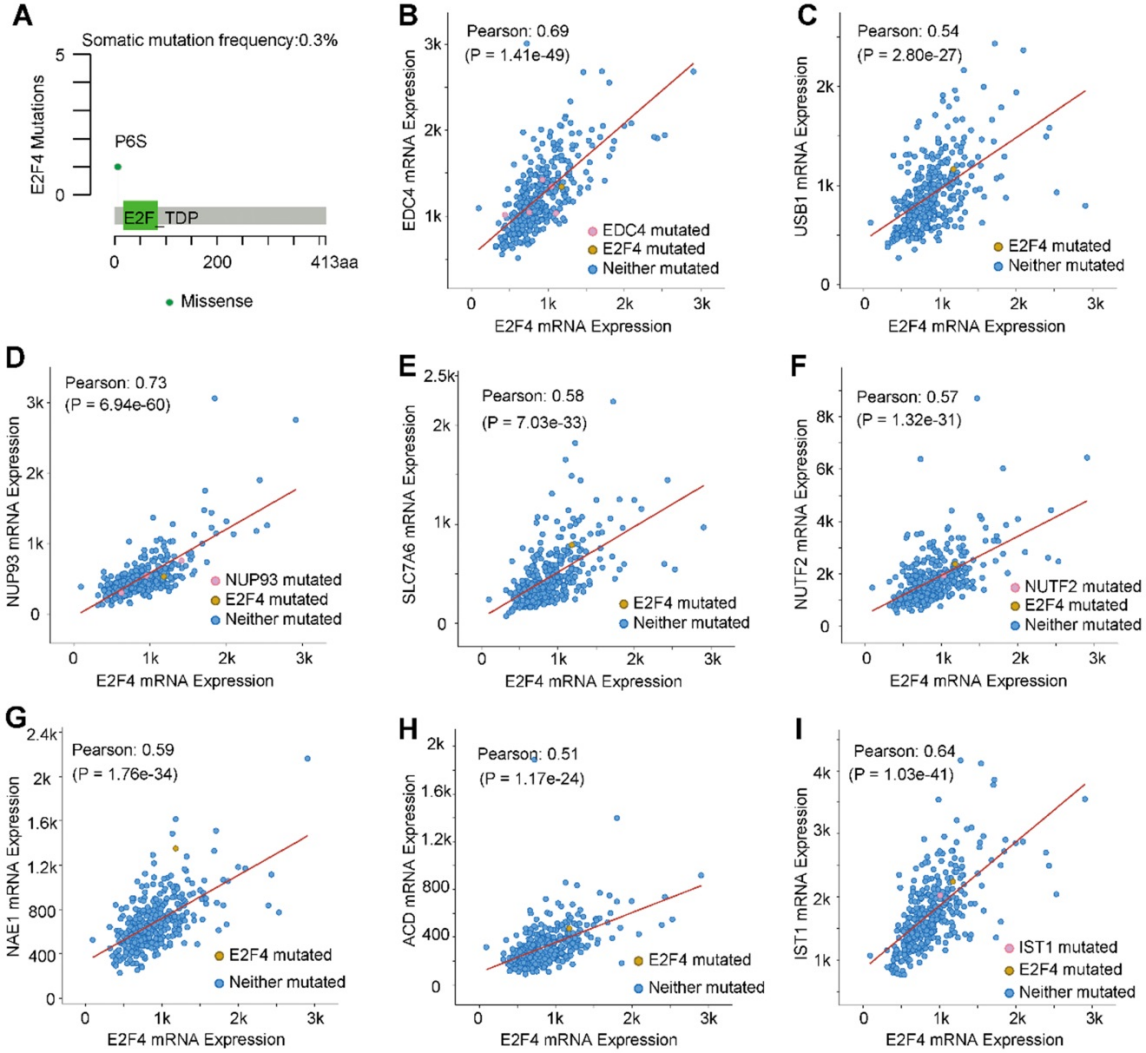
**E2F4 gene mutations and genes that are co-expressed in HCC. (a)** E2F4 gene somatic mutation frequency. **(b-i)** The relationship between a mutation in E2F4 and a mutation also occuring in (b) EDC4, (c) USB1, (d) NUP93, (e) SLC7A6, (f) NUTF2, (g) NAE1, (h) ACD, and (i) IST1. **Abbreviations:** HCC, hepatocellular carcinoma; Pearson, Pearson's correlation coefficient; EDC4, enhancer of mRNA decapping 4; USB1, U6 snRNA biogenesis phosphodiesterase 1; NUP93, nucleoporin 93; SLC7A6, solute carrier family 7 member 6; NUTF2, nuclear transport factor 2; NAE1, NEDD8 activating enzyme E1 subunit 1; ACD, adrenocortical dysplasia protein homolog; IST1, IST1 factor associated with ESCRT-III.

The mutation characteristics of E2F4 were further investigated, and an analysis using the cBioPortal was performed 28. Results indicated that the frequency of somatic mutations in E2F4 was 0.3% in HCC (**Figure 4A**). Moreover, the correlation between E2F4 expression with the mutations and co-expressed genes was explored. We obtained eight highly co-expressed genes in HCC based on the TCGA database (**Table 3**). As shown in **Figure 4B-4I**, E2F4 expression was significantly positively associated with the following genes: enhancer of mRNA decapping 4 (*EDC4;* Pearson correlation coefficient [PCC] 0.69, p = 1.41e-49), IST1 factor associated with ESCRT-III (*IST1*; PCC 0.64, p = 1.03e-41), nucleoporin 93 (*NUP93*; PCC 0.73, p= 6.94e-60), U6 snRNA biogenesis phosphodiesterase 1 (*USB*; PCC 0.54, p = 2.80e-27), solute carrier family 7 member 6 (*SLC7A6*; PCC 0.58, p = 7.03e-33), nuclear transport factor 2 (*NUTF2*; PCC 0.57, p = 1.32e-31), NEDD8 activating enzyme E1 subunit 1 (*NAE1*; PCC 0.58, p = 1.76e-34), and adrenocortical dysplasia protein homolog (*ACD*; PCC 0.51, p = 1.17e-24). These results provided the mutation characteristics of E2F4 and the distribution of the genes co-expressed with E2F4 in HCC, implying that E2F4 gene expression has close co-relationship with other genes expression.

### DEGs of E2F4 in HCC and selection of hub genes

**Figure 5:**
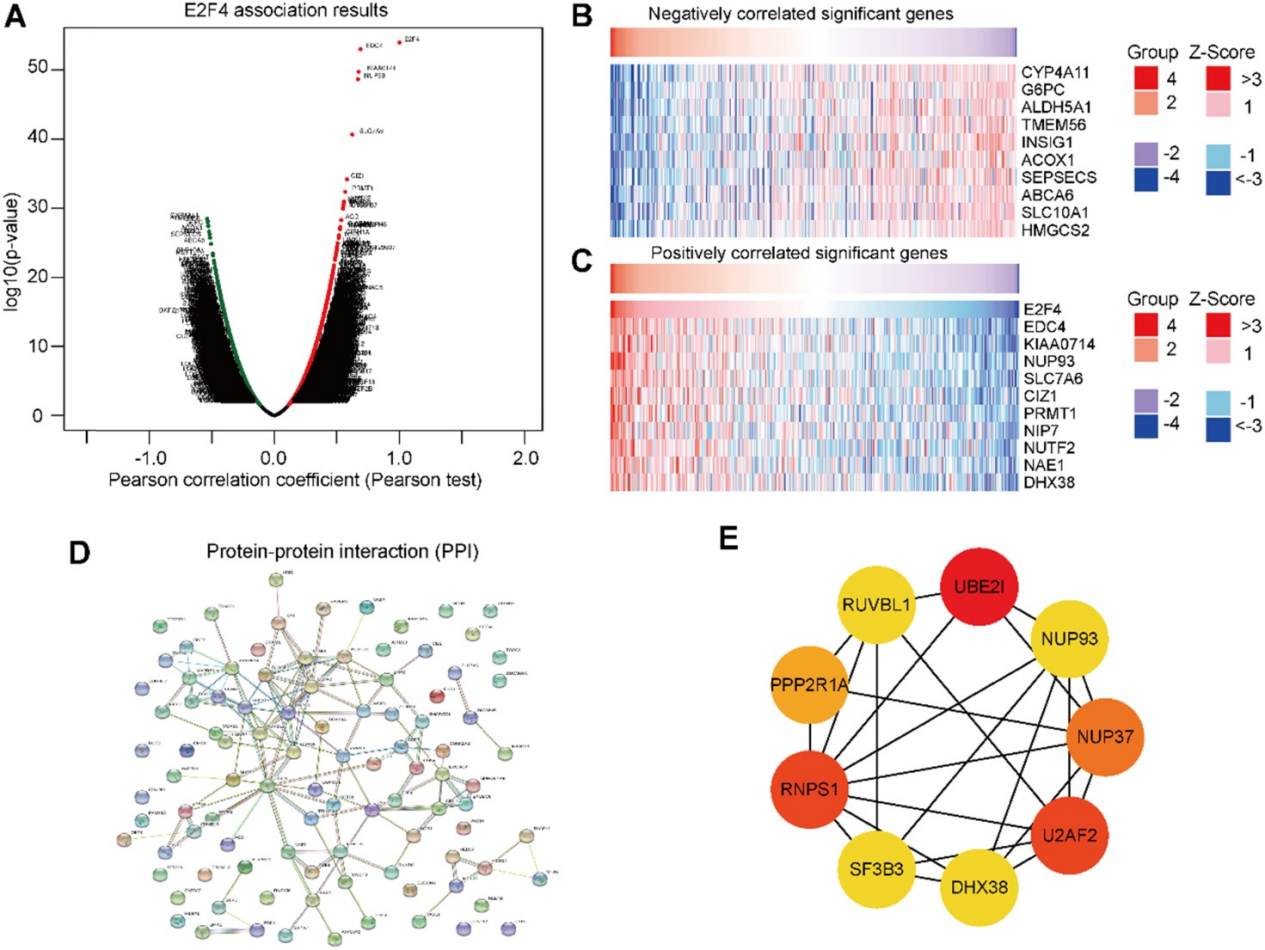
**Genes associated with E2F4 expression in HCC. (a)** Volcano plot showing the positively and negatively DEGs in TCGA. **(b-c)** The top 10 negatively and positively correlated genes with E2F4. **(d)** The PPI network of E2F4 expression. **(e)** The nine hub genes of E2F4. **Abbreviations:** HCC, hepatocellular carcinoma, DEGs, differentially expressed genes; TCGA, The Cancer Genome Atlas; PPI, protein-protein interaction. CYP4A11, specificity of cytochrome P450 4A11; G6PC, glucose-6-phosphatase (G6Pase) catalytic; ALDH5A1, Aldehyde dehydrogenase 5 family, member A1; INSIG1, insulin-induced gene 1; ACOX1, acyl Coenzyme A xidase 1; SEPSECS, spontaneous excitatory postsynaptic currents; ABCA6, ATP-binding cassette subfamily A member 6; SLC10A1 (NTCP), Na-taurocholate cotransporting polypeptide; HMGCS2, 3-hydroxy-3-methylglutaryl-CoA synthase 2; EDC4, Enhancer of mRNA decapping protein 4; NUP93, Nucleoporin 93; SLC7A6, CIZ1, Cip1-interacting zinc finger protein 1; PRMT1, protein arginine N-methyltransferase 1; NIP7, nodulin 26-like intrinsic protein7; NUTF2, nuclear transport factor 2; NAE1, NEDD8-activating enzyme 1; DHX38, DEAH-Box Helicase 38. RUVBL1, RuvB-like protein 1; UBE2I, ubiquitin-conjugating enzyme E2I, PPP2R1A, Protein Phosphatase 2 Scaffold Subunit Aalpha; RNPS1, RNA-binding protein with serine-rich domain 1; SF3B3, U2AF2, U2 small nuclear RNA auxiliary factor 2, NUP37, Nucleoporin 37.

To further validate the biological activities of E2F4 in HCC, the E2F4 DEGs were evaluated in HCC. The volcano map identified E2F4-related DEGs with positively related genes on the right of the plot and negatively related genes on the left of the plot (**Figure 5A**). Additionally, the heatmaps of the top 10 negatively related genes identified were *CYP4A11*, *G6PC*, *ALDH5A1*, *TMEM56*,* INSIG1*, *ACOX1*, *SEPSECS*, *ABCA6*, *SLC10A1*, and *HMGCS2* (**Figure 5B**). The top 10 positively related genes were *EDC4*,* KIAA0714*,* NUP93*,* SLC7A6*,* CIZ1*,* PRMT1*, *NIP7*,* NUF2*, *NAE1*, and *DHX38* (**Figure 5C**).

To determine the relationship of the top 100 positively related genes of E2F4 in HCC, a PPI network was established. As shown in** Figure 5D**, frequent interaction among the top 100 genes have close relationships with E2F4 expression. After calculating by degree using Cystoscope software, we obtained nine hub genes that revealed the closest relationships. The nine hub genes were *HDX38*, *NUP37*, *NUP93*, *PPP2P1A*, *RNPS1*, *RUVBL1*, *SF3B3*, *U2AF2*, and *UBE2IR* (**Figure 5E**). The expression of E2F4 was significantly associated with the expression of the hub genes (**Supplementary Figure 1**), especially for *SF3B3* (R = 0.81). Based on these results, the relationship between E2F4 and hub genes was a novel reason for tumor progression. Our results demonstrated that E2F4 expression was correlated with numerous crucial genes involved in many biological regulatory activities such as the cell cycle, protein modification, cellular transcription, and glycolipid metabolism.

### E2F4 related pathways

**Figure 6:**
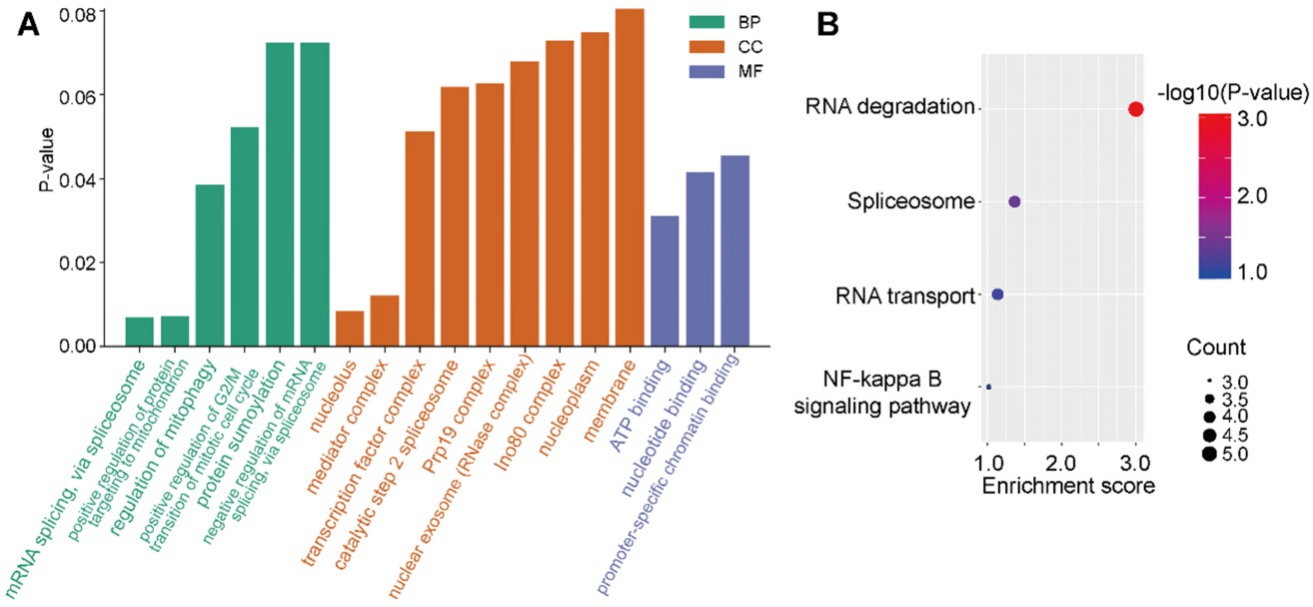
**Distribution of E2F4 associated pathways. (a)** Significantly enriched GO terms associated with BP, CC, and MF annotations in the TCGA-LIHC cohort. **(b)** Enrichment analysis of the E2F4 related pathway, where red indicates a significantly positively correlated pathways. **Abbreviations:** GO, gene ontology; BP, biological process; CC, cellular component; MF, molecular function; TCGA-LIHC, The Cancer Genome Atlas - liver hepatocellular carcinoma.

To investigate the regulation of signaling pathways associated with E2F4, the top 100 significantly related genes were used in KEGG and GO pathway enrichment analyses. GO results revealed six significant biological processes (BP), nine cellular components (CC), and three molecular functions (MF; **Figure 6A**). More specifically, E2F4-related pathways involved in BPs included mRNA splicing via spliceosome, regulation of mitophagy, and positive regulation of protein targeting the mitochondrion. Negative regulation of mRNA splicing via spliceosome revealed that E2F4 actively participates in nucleic acid, mitochondria, and accounting-related protein regulation. In CC analysis, the nucleolus, mediator complex associated pathways were significantly correlated with E2F4 function. In the MF subgroup, nucleotide binding, promoter-specific chromatin binding, and ATP binding pathways indicated significantly positive affiliation to E2F4 gene regulation (**Figure 6A**). Moreover, according to KEGG analysis, RNA degradation suggested that the E2F4 gene plays a crucial role in cellular biology and the E2F4 gene was positively associated with RNA degradation and negatively associated to RNA transport and the spliceosome (**Figure 6B**). These results collectively demonstrated that E2F4 has a wide range of effects on the production of the global transcriptome and the regulation of genes and pathways involved in mitochondrial metabolism.

### E2F4 affects tumor purity, immune infiltration, and the immune signature of HCC

**Figure 7:**
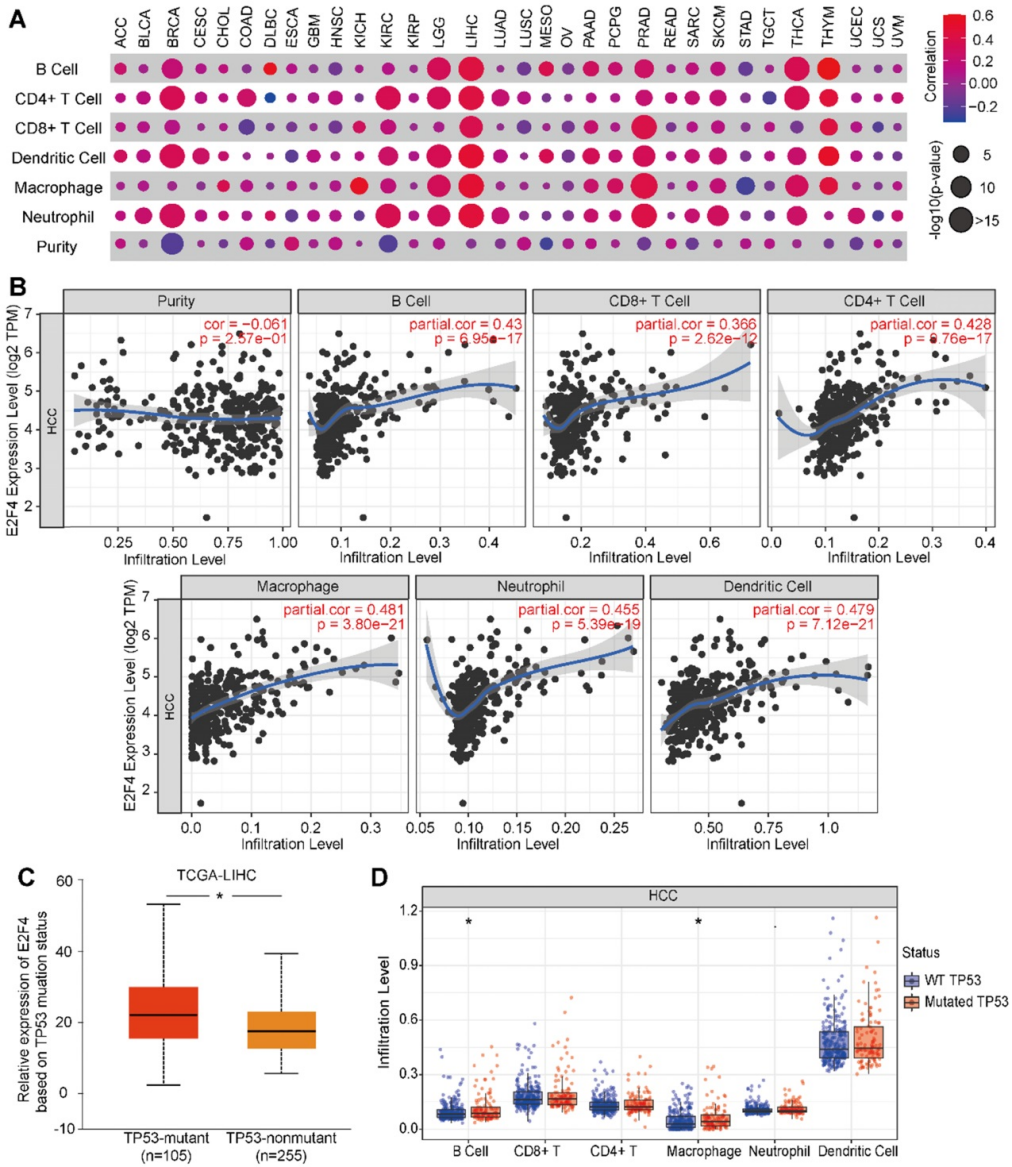
**The relevance of E2F4 gene expression and immune infiltration of the HCC cohort in the TIMER database. (a)** The correlation of E2F4 expression and immune cell infiltration across 32 solid cancers. **(b)** Correlation of immune purity with E2F4 gene expression. Immune infiltration levels in HCC of B cells, CD4^+^ T cells, CD8^+^ T cells, neutrophils, macrophages, and dendritic cells. **(c)** E2F4 expression correlated with TP53 mutation. **(d)** The affect of TP53 mutation on the infiltration of the immune cells. **Abbreviations:** HCC, hepatocellular carcinoma; TIMER, Tumor Immune Estimation Resource; TP53, tumor protein p53.

Next, we evaluated if E2F4 expression was associated with tumor immune status across 32 solid cancer types. E2F4 expression was found to be significantly associated with immune cell infiltration among several cancers, especially in HCC (**Figure 7A**). In HCC, E2F4 was positively correlated with immune cell infiltration by cells including B cells (partial. cor = 0.43, p = 6.95e-17), CD4^+^ T cells (partial. cor = 0.428, p = 0.76e-17), CD8^+^ T cells (partial. cor = 0.366, p = 2.62e-12), macrophages (partial. cor = 0.481, p = 3.80e-21), neutrophils (partial. cor = 0.455, p = 5.39e-19), and dendritic cells (partial. cor = 0.479, p = 7.12e-21). However, E2F4 expression was negatively related to immune purity (**Figure 7B**). Considering the high frequent of TP53 mutations in HCC, we investigated the effect of mutated TP53 on E2F4 expression. As shown in **Figure 7C**, we observed that E2F4 expression was elevated in the TP53-mutant group (n = 105) compared with the TP53 non-mutant group (n = 255). The TP53 mutation status was further evaluated regarding the level of immune cell infiltration. In this study, it was discovered that B cells and macrophages were increased with TP53 mutation compared to wide-type TP53 (**Figure 7D**). Our results demonstrated that TP53 mutation might play an essential regulatory role in tumor immune cell infiltration.

## Discussion

HCC is a worldwide threat with significant morbidity and mortality based on the stage of the disease at presentation 29. Current treatment strategies such as resection, chemotherapy, radiation, immunotherapy, and gene therapy are improving, but recurrence of illness due to therapy resistance results in high mortality. And the five-year recurrence rate after resection is up to 60% to 70% 30. Most HCC patients suffer from a clinically poor prognosis 31. Therefore, it is urgent to find out effective prognostic biomarkers and comprehensively acknowledged the genomic associated characteristics to predict the prognosis of patients with HCC before resection. Hence, the selection of optimum candidate genes for early detection and targeted therapy is especially important. The origin of HCC is a multistep process with a highly progressive accumulation of molecular transcriptomic alterations 32. Members of the E2F family are crucial factors in tumorigenesis. Studies have reported that these transcription factors regulate multiple cytokine genes and signaling pathways that are involved in viral tumorigenesis progression 33. For example, a recent study showed that E2F1 could regulate the expression of DEAD/H-Box helicase 11 (DDX11) to promote tumor progression in HCC 34. Importantly, E2F4 has been revealed to be a critical regulator of HCC 35.

In this study, we observed that E2F4 expression was dysregulated in pan-cancer, and especially in HCC. Our study has provided a more detailed picture of E2F4 expression in relation to clinical characteristics, prognosis, immune cell infiltration, hub genes, and pathway crosstalk in HCC. Our results demonstrated that E2F4 was highly expressed in HCC, and Kaplan-Meier analyses indicated that E2F4 could be a potential prognostic factor in HCC. Similar to our results, Khaleel and colleagues reported that E2F4 could serve as an outcome prognosis predictor for breast cancer 36. Another study demonstrated that E2F4 played an essential role in the drug-sensitivity and tumor progression of epithelial ovarian cancer 37. These findings showed that E2F4 could serve as a novel biomarker for cancer diagnosis and prognosis prediction.

A network analyst algorithm was utilized to further explore the relationship between E2F4 and co-expressed genes, and the crucial enriched pathways involved in this crosstalk. We found that E2F4 could be identified as a regulator of hub genes in liver cancer. In support of our results, studies have demonstrated that E2F4 function as crucial regulator in liver cancer 38. The increased E2F4 in breast cancer is also associated with breast cancer liver metastasis 39. it is also estimated that E2F4 acts as bridging genes in the process of colorectal cancer liver metastasis 40. Additionally, it was revealed that E2F4 could be a hub node regulatory gene in the subnetwork of HCC. Collectively, these findings suggest that E2F4 may play a critical regulatory role in HCC.

The tumor microenvironment has a strong influence on the carcinogenesis of HCC 41. The underlying mechanisms of liver immune tolerance are still mostly unknown. Genetic diversity and microenvironment have a close relationship with HCC progression 42. Therefore, we aimed to expand our current knowledge regarding the E2F4 gene in immune response regulation, taking into consideration the tumor purity and tumor immunity. We found that immune cells have high immune cell purity in HCC. The B cells, CD8^+^ T cells, CD4^+^ T cells, macrophages, neutrophils, and dendritic cells are co-related immune cells, which serve critical roles in HCC immune infiltration. Altogether, these data powerfully indicate that E2F4 could be a crucial factor mediating immune-associated pathways.

To further investigate the signaling crosstalk involved in controlling abnormal E2F4 gene expression, we examined the E2F4 co-expression network. Our study validates that E2F4 influences cell cycle regulation, DNA repair, and replication. At the same time, E2F4 is also active in metabolic associated process, such as mRNA splicing via the spliceosome and regulation of mitophagy, positive regulation of protein targeting to the mitochondrion, nucleolus, mediator complex ATP binding, nucleotide, binding promoter-specific chromatin binding processes. These molecular activities are consistent with tumor carcinogenesis, and E2F4 could participate in tumor progression via the regulation of these pathways.

Taken together, these findings demonstrate that the up-regulation of E2F4 could be a promising molecular target for the diagnosis and treatment of HCC. Reasonably, the potential role of E2F4 in immune environment regulation and clinical conditional diagnostic function still requires further validation.

## Supplementary Material

Supplementary figure S1.

## Figures and Tables

**Table 1:** Correlation between E2F4 expression and clinicopathological characteristics of patients with HCC

	Variables	E2F4 expression		Total	χ^2^	p value
		Low	High			
**Age (years)**					0.262	0.608
	≤ 50	15	25	40		
	> 50	21	28	49		
**Sex**					1.116	0.291
	Female	2	8	10		
	Male	34	45	79		
**Grade**					0.597	0.44
	1/2	22	28	50		
	3	14	25	39		
**TNM stage**					0.187	0.665
	I	26	36	62		
	II/III	10	17	27		
**Cirrhosis**					0	1
	Negative	4	5	9		
	Positive	32	47	79		
**AFP**					1.154	0.283
	Negative	18	20	38		
	Positive	18	32	50		
**PD-L1**					4.751	0.029
	Negative	13	11	24		
	Positive	17	42	59		

Abbreviations: HCC, hepatocellular carcinoma; TNM, tumor node metastasis; AFP, alpha-fetoprotein; PD-L1, programmed death-ligand 1.

**Table 2:** Univariate and multivariate analyses of the factors correlated with overall survival of patients with HCC

	Univariate analysis				Multivariate analysis		
	HR	95% CI	p value		HR	95% CI	p value
**Age**	1.383	0.753-2.54	0.296				
**Sex**	1.438	0.445-4.652	0.544				
**Grade**	1.814	0.999-3.293	0.05		1.74	0.948-3.194	0.074
**TNM stage**	1.783	1.045-3.043	0.034		1.655	0.949-2.885	0.076
**E2F4 expression**	2.071	1.096-3.917	0.025		2.121	1.119-4.023	0.021

Abbreviations: HCC: hepatocellular carcinoma; HR, hazard ratio; CI, confidence interval; TNM, tumor node metastasis.

**Table 3:** Top eight genes co-expressed with E2F4.

Correlated Gene	Cytoband	Spearman's Correlation	p value
EDC4	16q22.1	0.697	6.90E-52
IST1	16q22.2	0.655	5.47E-44
NUP93	16q13	0.654	8.89E-44
SLC7A6	16q22.1	0.634	1.44E-40
NUTF2	16q22.1	0.576	4.04E-32
NAE1	16q22.1	0.568	3.74E-31
ACD	16q22.1	0.567	5.84E-31
USB1	16q21	0.566	7.31E-31

Abbreviations: EDC4, enhancer of mRNA decapping 4; IST1, IST1 factor associated with ESCRT-III; NUP93, nucleoporin 93; SLC7A6, solute carrier family 7 member 6; NUTF2, nuclear transport factor 2; NAE1, NEDD8 activating enzyme E1 subunit 1; ACD, adrenocortical dysplasia protein homolog; USB1, U6 snRNA biogenesis phosphodiesterase 1.
