# Supplementary material for: Transcription factor E2F4 is an indicator of poor prognosis and is related to immune infiltration in hepatocellular carcinoma
Source: J Cancer. 2021 Jan 21;12(6):1792–803. doi: 10.7150/jca.51616 (PMC7890309; doi:10.7150/jca.51616)

Supplementary Figure 1. The relevance of E2F4 gene expression in relation to the nine hub genes. The correlation between E2F4 expression and (**a**) NUP93, (**b**) NUP37, (**c**) PPP2R1, (**d**) RNPS1, (**e**) RUVBL, (**f**) SF3B3, (**g**) U2AF2, (**h**) UBE2I, and (**i**) DHX38 in HCC. Abbreviations: NUP93, nucleoporin 93; NUP37, nucleoporin 37; PPP2R1, protein phosphatase 2 regulatory subunit A; RNPS1, RNA-binding protein with serine-rich domain 1; RUVBL, RuvB-like AAA ATPase 1; SF3B3, splicing factor 3B subunit 3; U2AF2, U2 small nuclear RNA auxiliary factor 2; UBE2I, ubiquitin conjugating enzyme E2 I; DHX38, DEAH-box helicase 38.

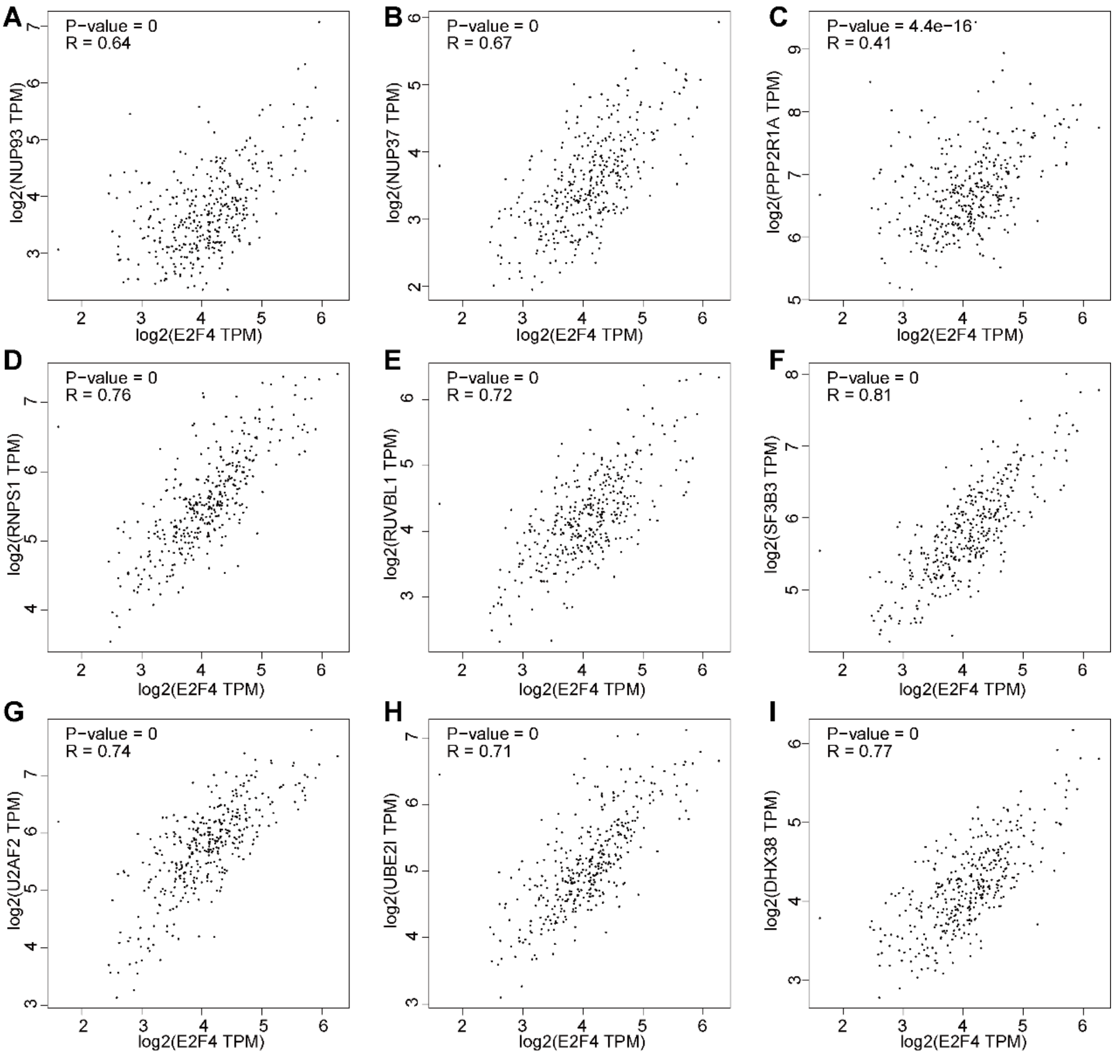

Supplement: Supplementary file 1 — Supplementary figure S1. [file jcav12p1792s1.pdf]
